# The effect of scheduled metamizole on opioid consumption after cardiac surgery

**DOI:** 10.3389/fphar.2026.1767338

**Published:** 2026-02-18

**Authors:** Chen Seidenberg, Adina Grunberger, Ruth Mishali, Avi Hefets, Pierre Singer, Eric Setton, Michal Slevin Kish

**Affiliations:** 1 Herzliya Medical Center, Herzliya, Israel; 2 The research laboratory on Computerized Decision Making, Dina Recanati School of Medicine, Reichman University, Herzliya, Israel

**Keywords:** cardiac surgeries, metamizole, multimodal analgesia, opioid analgesics, pain management, postoperative care

## Abstract

This retrospective study evaluates the impact of implementing a standardized scheduled metamizole dosing protocol within a multimodal analgesia approach after cardiac surgery. The results showed that scheduled metamizole administration was associated with lower opioid consumption, while maintaining adequate pain control and safety. Pain scores measured by the Numeric Rating Scale improved from 1.12 pre-protocol to 0.89 post-protocol (p < 0.0001). Mean opioid consumption decreased from 119.51 mg morphine equivalents to 95.91 mg (p < 0.0001). No cases of clinically relevant agranulocytosis or persistent neutropenia were observed. Renal function, assessed by changes in serum creatinine, showed no significant differences between groups, suggesting renal safety. Despite improved analgesia and reduced opioid use, hospital length of stay increased slightly, potentially due to confounding factors. Our findings support scheduled metamizole as a safe and effective opioid-sparing agent in postoperative cardiac surgery pain management. Further prospective randomized trials are warranted to confirm these results and establish optimal protocols.

## Introduction

Effective postoperative pain management is critical for optimizing recovery following cardiac surgery. Inadequate analgesia is linked to impaired respiratory mechanics and adverse hemodynamic responses (e.g., hypertension, tachycardia) and to pulmonary complications such as atelectasis and pneumonia; poorly controlled pain can delay functional recovery and is associated with persistent postsurgical pain. Contemporary guidance endorses multimodal, opioid-sparing strategies in cardiac surgery as a core element of enhanced recovery after surgery (ERAS) and opioid-sparing strategies (Echeverria-Villalobos et al., 2020; Grant et al., 2023; Loria et al., 2022; Othenin-Girard et al., 2025). Multimodal analgesia has emerged as an essential component of ERAS cardiac recommendations. Optimizing postoperative pain control includes concurrent use of primarily non-opioid analgesics around the clock in scheduled dosing. This strategy reduces reliance on opioids, as the medications have additive, if not synergistic, analgesic effects.

Scheduled around the clock dosing of non-opioids often provides better postoperative pain control compared to pro re nata (PRN) administration in several surgical populations (Blitz et al., 2022; Inoue et al., 2021). However, in routine practice, PRN administration remains common, and implementation of standardized multimodal care is heterogeneous (Lui et al., 2023; Yoon et al., 2025). Metamizole (dipyrone, Optalgin®) is widely used in many countries as a non-opioid analgesic, authorized in the European Union for moderate to severe pain. Oral paracetamol is FDA-approved for the temporary relief of mild to moderate pain. This distinction in labeled pain indications aligns with differences in clinical utilization patterns and pharmacologic classification (FDA, 2025; EMA, 2025; Erlenwein, 2016). The existing literature demonstrates that metamizole provides adequate analgesia comparable to NSAIDs and is non-inferior—and in some surgical contexts, occasionally superior—to paracetamol for postoperative pain control (Erlenwein, 2016; Jeyaraman et al., 2024). In a recently randomized coronary artery bypass graft (CABG) trial, scheduled metamizole reduced the need for additional co-analgesics without attenuating aspirin’s antiplatelet effect (Luz et al., 2025; Petermichl et al., 2025).

Safety considerations remain primary priority, as cardiac surgery itself triggers a marked inflammatory response—locally within the pericardial space and systemically. This is most pronounced in the first 24–48 h after cardiac pulmonary bypass (CPB), which can be accompanied by leukocytosis/neutrophilia (Squiccimarro et al., 2022; El-Diasty et al., 2024).

Cardiac surgery associated acute renal failure (CSA-AKI), usually evolves within the first 48–72 h, and is associated with short and long adverse outcomes (Scurt et al., 2024a; Lindhardt et al., 2024).

Concerning hematologic safety, metamizole-associated agranulocytosis is very rare but life-threatening side effect, which require awareness and vigilance during treatment (patient education and prompt complete Blood Count (CBC) testing if systemic symptoms like high fever, chills, sore throat, difficulties swallowing occur) (Chatzimanouil et al., 2023; European Medicines Agency, 2024; Robich, 2020). Evidence on renal safety relative to other NSAIDs is mixed and context-dependent (Scurt et al., 2024a; Chatzimanouil et al., 2023).

This retrospective real-world study evaluated scheduled versus PRN metamizole use as part of a postoperative multimodal analgesia protocol in post cardiac surgery patients. Primary outcomes were pain intensity, opioid consumption by morphine milligram equivalents (MME), and safety parameters [white blood cells (WBC)/neutrophils and serum creatinine], to determine optimal metamizole use in this population.

## Materials and methods

### Study design and setting

This is a single center, retrospective, observational cohort study conducted between 03.2019 and 01.2025 at the Herzliya Medical Center. The study assessed the impact of changing postoperative multimodal analgesia protocol from PRN dosing of metamizole to “around the clock” administration, in adult patients undergoing cardiac surgery. The protocol was implemented hospital wide in August 2023. Clinical data were extracted from electronic medical records for all eligible patients treated between March 2019 and January 2025. To ensure patient confidentiality and comply with data protection regulations, all extracted data were anonymized by removing personal identifiers and replacing them with unique study codes before analysis.

Data handling procedures followed institutional policies for secure storage and limited access by authorized personnel only.

### Study population

All adult patients (≥18 years old) who underwent cardiac surgery between March 2019 and January 2025 were screened for eligibility. Surgical procedures included CABG, isolated or combined valve replacement or repair, thoracic aortic surgery, and other complex cardiac operations. Patients were excluded if they were hospitalized for fewer than three postoperative nights, had contraindications to metamizole (e.g., known hypersensitivity) or did not have data on pain scores. A total of 2,234 patients met the inclusion criteria and were stratified into two cohorts: pre-protocol (n = 1,606) and post-protocol (n = 628).

### Postoperative analgesia protocol

Prior to protocol implementation, postoperative patients’ pain was treated with a combination of controlled release oxycodone 10 mg twice daily for 3 days and scheduled around the clock every 8 h dosing of paracetamol 1000 mg without stopping date.

**FIGURE 1:**
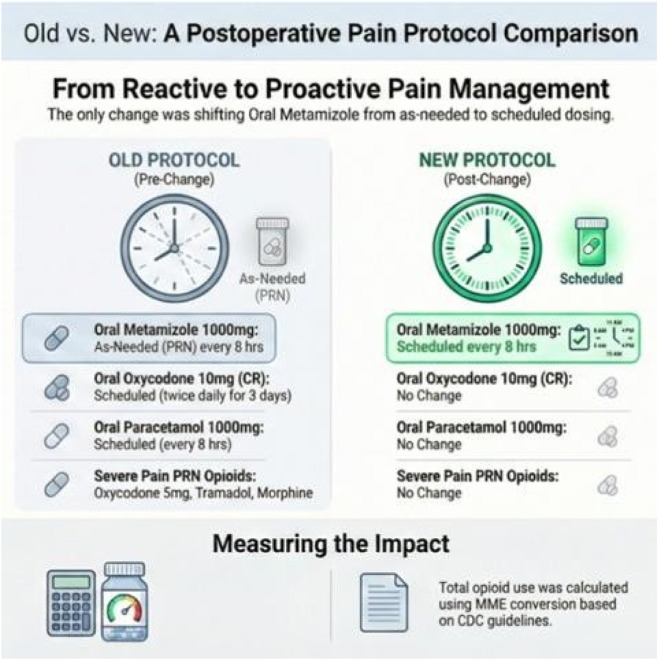
Postoperative pain management before and after protocol implementation.

For acute moderate-severe pain metamizole 1000 mg was prescribed as a PRN medication, while immediate release oxycodone 5 mg or IV tramadol 1000 mg or morphine 2 mg were prescribed as PRN options for acute severe pain. Following the protocol change, metamizole was prescribed and administered as around the clock medication alongside with paracetamol, on a fixed schedule of 1 g every 8 h, unless contraindicated (Figure 1).

Total opioid consumption per patient was calculated by MME conversion ratio based on centers for disease control and prevention (CDC) guidelines standard equianalgesic conversion tables (Dowell et al., 2022).

## Outcomes

### Pain assessment

Pain was assessed as a continuous variable using the NRS (0–10). Analyses included comparison of overall mean pain scores and subgroup analysis of mean NRS values among patients with moderate to severe pain (defined as NRS 7–10). Pain scores were extracted from routine clinical documentation, which was incomplete and irregular, reflecting variability in documentation practices typical of real-world clinical settings. All available data were included in the analysis. Proportions of patients across pain severity categories were calculated as a descriptive measure. The primary pain outcomes were the overall mean NRS score and the mean NRS score within the moderate to severe pain subgroup, both calculated across all available assessments during hospitalization.

#### Analgesic utilization

Metamizole use was recorded as the total daily dose (mg/patient). Total opioid consumption was calculated as the cumulative MME administered throughout the entire hospital stay. Opioids were administered according to the world health organization (WHO) analgesic ladder approach (Anekar and Cascella, 2023), based on the three steps of mild to severe pain treatment.

### Laboratory parameters

Renal function was assessed using serum creatinine levels obtained from routine clinical testing. Potential renal effects associated with metamizole administration were evaluated by assessing changes in serum creatinine from baseline on postoperative days 4 and 7, ensuring sustained drug exposure while minimizing the influence of transient perioperative renal fluctuations commonly observed after cardiac surgery. The selected assessment timeframe is also consistent with prior studies examining the pharmacologic effects and adverse event profile of metamizole (Bachmann et al., 2021). Indications for dialysis followed clinical guidelines. All dialysis cases were reviewed manually to determine the underlying clinical indication, regardless of specific creatinine values. Hematologic safety was evaluated using CBCs. Neutropenia was defined as absolute neutrophilia count <1,500/µL.

### Length of stay

Length of hospital stay was calculated from operative day (POD0) to the day of discharge and compared between pre- and post-protocol groups.

### Statistical analysis

Comparisons between the pre-protocol (March 2019–July 2023) and post-protocol (August 2023–December 2024) cohorts were performed using standard tests appropriate for data type and distribution. Categorical variables are presented as counts and percentages, and continuous variables as means ± standard deviation (SD). Normality was assessed with the Shapiro–Wilk test. Between-group comparisons used independent-samples t-tests for normally distributed.

### Laboratory parameters

Variables and Mann–Whitney U tests for non-normal or ordinal data (e.g., NRS pain scores). Laboratory results were analyzed using delta (Δ) changes between postoperative days 4 and 1 and between 7 and 1. Two-tailed p < 0.05 was considered statistically significant, and 95% confidence intervals were reported where relevant. Analyses were performed using standard validated statistical software.

### Ethical considerations

This study was approved by the institutional Helsinki Committee (IRB), approval number [0002- 23-HMC]. Given the retrospective nature of the study and use of anonymized data, the requirement for informed consent was waived.

## Results

### Study population

A total of 2,234 patients undergoing cardiac surgery were included (pre-protocol, n = 1,606; post-protocol, n = 628). Baseline demographics are detailed in Table 1.

**TABLE 1:** Baseline demographic and clinical characteristics of study participants, stratified by protocol group.

Baseline demographic of participants	Before protocol implementation	After protocol implementation
Male	1,259	499
Females	347	129
Mean age	65.169	65.170
Surgery type		
CABG	61.8%	60.2%
Aortic valve replacement	15.6%	16.6%
Mitral valve replacement	9.4%	10.2%
CABG + valve	7.9%	6.4%
Thoracic aorta	1.6%	3.2%
Tricuspid valve replacement\repair	1.4%	1.9%
Other	2%	1.6%

#### Pain outcomes

**FIGURE 2:**
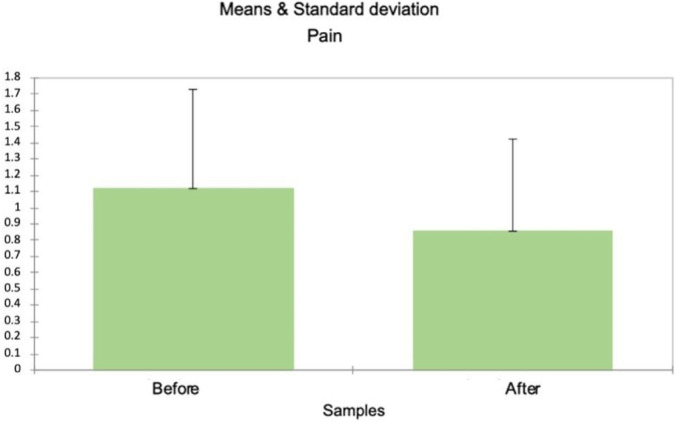
Pain intensity (NRS 0-10) before and after protocol implementation.

The results showed that scheduled metamizole administration was associated with lower opioid consumption, while maintaining adequate pain control and safety (Figure 2).

The mean NRS decreased from 1.121 [SD = 1.949] pre-protocol to mean = 0.89 [SD = 1.901] post-protocol (Mann–Whitney U = 239,251,492, p-value <0.0001). However, no significant difference was observed for moderate to severe pain (NRS 7–10; U = 223,199; p-value = 0.853). The proportion of patients reporting NRS 0–3 increased following protocol implementation (64% → 73.1%), while moderate–severe pain (NRS ≥4) remained low and similar between groups (13% vs. 10.8%), as summarized in Table 2.

**TABLE 2:** Distribution and intensity of postoperative pain scores (NRS 0–10) in pre- and post-protocol cohorts.

Postoperative pain scores	Before protocol implementation	After protocol implementation
0	64%	73.15%
1–3	23%	16.03%
4–6	10%	7.38%
7–10	3%	3.44%

#### Analgesic utilization

Total scheduled metamizole dosing per patient during the hospitalization period (average 5 days) markedly increased consumption (Pre: mean = 3.468 g per patient, SD = 3.542; Post: mean = 16.205 g per patient, SD = 9.636; independent t-test p-value <0.001).

#### Opioid use (MME)

**FIGURE 3:**
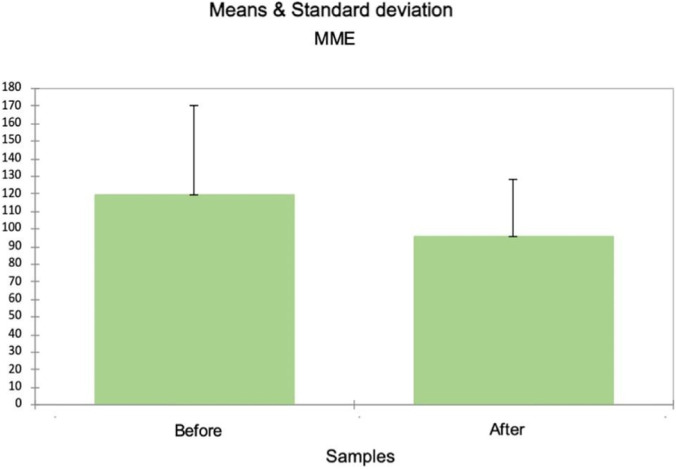
MME before and after protocol implementation.

MME per patient decreased substantial post-protocol (Figure 3), from mean = 119.51 mg [SD = 51.086] pre protocol, to mean = 95.914, [SD = 32.648 mg] post protocol; independent t-test p-value <0.0001).

### Laboratory markers

Serum creatinine changes measured by the delta (Δ) postoperative day 4 minus day 1, showed no significant difference between groups (pre-protocol: mean = 0.092 [SD = 0.529 mg/dL], n = 999; post-protocol: mean = 0.149 [SD = 0.594 mg/dL] n = 422; mean difference 0.057; 95% CI [−0.119, 0.006]; p-value = 0.074). Similarly, the delta (Δ) postoperative day 7 minus day 1was comparable (pre: mean = 0.072, SD = 0.691 mg/dL, n = 242; post: mean = 0.094, SD = 0.613 mg/dL, n = 141; mean difference −0.022; 95% CI [−0.160, 0.116]; p-value = 0.752). Dialysis indication followed clinical guidelines. Six patients required dialysis, attributed to chronic kidney disease or post-CPR complications. Patients with leukocyte counts <2 × 10^9^/L underwent manual neutrophil review, revealing no agranulocytosis or persistent cytopenias. Most neutropenia episodes (9 patients) were isolated, transient, or attributed to lab errors; counts normalized within a day.

#### Length of stay

Hospital stay durations for the pre-and post-protocol groups are summarized in Table 3. Overall hospital stay increased slightly post-protocol (pre: mean = 10.448, SD = 8.411; post: mean = 11.209, SD = 7.683. The independent t-test indicated a statistically significant increase of 0.76 nights (95% CI [-0.62, −0.90]; t = −10.65; p-value <0.0001).

**TABLE 3:** Hospital duration for study participants before and after protocol implementation.

Hospital duration	Before protocol implementation	After protocol implementation
Total nights observations	40,382	19,404
Minimum	3	3
Maximum	50	46
Mean	10.448	11.209
SD	8.411	7.683

## Discussion

This retrospective study evaluated the impact of implementing a standardized scheduled around the clock metamizole dosing protocol as part of a multimodal analgesia approach following cardiac surgery. Our findings demonstrate that scheduled metamizole administration was associated with lower opioid consumption, while maintaining adequate pain control and preserving renal and hematologic safety.

### Context within enhanced recovery after surgery (ERAS)

ERAS protocols have emerged as a paradigm shift in perioperative cardiac care, emphasizing multimodal pain management that combines complementary medications and techniques to spare opioids and improve symptomatic and functional recovery (Bignami et al., 2020; Li et al., 2018). However, there remains limited data on the optimal use and effectiveness of individual non-opioid pharmacologic agents in cardiac surgery populations (Gregory et al., 2024). Despite mounting evidence supporting metamizole opioid-sparing effects and analgesic efficacy, and its widespread use in many countries, it is not routinely included in standardized ERAS protocols owing to regulatory restrictions and safety concerns—particularly regarding agranulocytosis risk—which led to its withdrawal from the United States and several other countries (Makkad et al., 2025; Andrade, 2020). Current ERAS non-opioid analgesic regimens typically include paracetamol, NSAIDs, ketamine, and dexmedetomidine, while metamizole is rarely mentioned and its use remains experimental or institution-dependent (Gregory et al., 2024). In this context, metamizole represents a potentially valuable addition or alternative for patients in whom traditional non-opioid analgesics are contraindicated, particularly given its established efficacy and relatively favorable safety profile in appropriate clinical settings.

Although metamizole was used in both groups, maximizing its use with scheduled around-the- clock administration of 1,000 mg three times daily led to a substantial reduction in mean NRS scores, from 1.121. (SD = 1.949) pre-protocol to 0.89 (SD = 1.901) post protocol (Mann–Whitney U = 239,251,492, p < 0.0001). This aligns with prior reports demonstrating that scheduled around-the-clock analgesia often provides better pain control compared to PRN administration in the early postoperative phase (Blitz et al., 2022; Inoue et al., 2021). Our data confirms these findings in the specific context of cardiac surgery, where inadequate analgesia is particularly detrimental due to its adverse effects on respiratory mechanics and hemodynamics (Echeverria-Villalobos et al., 2020; McDonall et al., 2025). Notably, while mean pain scores and opioid consumption decreased overall, no significant difference was found in the moderate-to-severe pain subgroup (NRS 7–10), (U = 223,199; p = 0.853). These findings may reflect the distinct impact of metamizole on different pain levels.

The concomitant reduction in opioid consumption (measured in morphine milligram equivalents) supports the efficacy of scheduled metamizole as an opioid-sparing agent. Mean MME per patient decreased from 119.51 mg (SD = 51.086) pre-protocol to 95.91 mg (SD = 32.648) post-protocol (p < 0.0001), representing a meaningful reduction. This is clinically important given the recognized risks of opioid-related adverse effects, dependence, and the broader implications of opioid sparing in cardiac surgery (Rauseo et al., 2025; Guimarães-Pereira et al., 2016). Our results echo findings from recent systematic reviews and meta-analyses indicating that metamizole provides adequate analgesia comparable to NSAIDs and paracetamol for postoperative pain control, particularly in intensive care settings (Luz et al., 2025; Dizner-Gołąb et al., 2025). While not directly evaluated in this study, multimodal opioid-sparing approaches are associated with reduced nausea and vomiting, decreased risk of delirium and falls, and lower risk of persistent opioid use and dependence following cardiac surgery (Grant et al., 2020; Aguerreche et al., 2021; Guinot et al., 2019; Fleming et al., 2016; Bignami et al., 2018). These benefits underscore the importance of maximizing the use of effective and safe non-opioid analgesics in comprehensive pain management protocols, particularly in populations where traditional options may be contraindicated.

### Safety considerations

#### Hematologic safety

The safety profile of scheduled metamizole was reassuring in this real-world cohort. Despite concerns about metamizole-associated agranulocytosis, no cases of clinically relevant agranulocytosis or persistent neutropenia were observed, consistent with recent regulatory and epidemiological reviews emphasizing the rarity of this adverse event when appropriate vigilance is maintained (European Medicines Agency, 2024; Tomidis Chatzimanouil et al., 2023). Instances of transient neutropenia observed were most likely attributable to laboratory variability given their brief duration and complete normalization in subsequent testing. The inflammatory response induced by cardiac surgery, characterized by leukocyte and neutrophil elevations peaking at 48 h and persisting up to 21 days postoperatively, may complicate the interpretation of hematologic parameters but did not obscure clinically relevant adverse events in our cohort (Lako et al., 2015). These findings suggest that with appropriate monitoring and awareness, scheduled metamizole can be safely administered in the acute postoperative period following cardiac surgery.

#### Renal safety

Renal safety remains a paramount concern given the known susceptibility of cardiac surgery patients to acute kidney injury (AKI), which affects a substantial proportion of this population and is associated with increased mortality and prolonged stays (Scurt et al., 2024a; Lindhardt et al., 2024).

Our analysis revealed no statistically significant differences in serum creatinine changes between pre- and post-protocol groups: Day 4 minus Day 1: Δ = 0.092 vs. 0.149 mg/dL, mean difference = 0.057; 95% CI [−0.119, 0.006]; p = 0.074. Day 7 minus Day 1: Δ = 0.072 vs. 0.094 mg/dL, mean difference = −0.022; 95% CI [−0.160, 0.116]; p = 0.752. These findings suggest that scheduled metamizole dosing did not exacerbate renal dysfunction. This is particularly relevant given the known nephrotoxic potential of NSAIDs and the overall renal vulnerability of cardiac surgery patients (Wang and Bellomo, 2017; Scurt et al., 2024b). While longer-term renal outcomes were not assessed in this study, our data support the relative renal safety of metamizole in the acute postoperative period, consistent with evidence suggesting a more favorable renal profile compared to traditional NSAIDs (Kötter et al., 2015). This characteristic may make metamizole particularly valuable in cardiac surgery populations, where renal preservation is a critical priority and NSAID use is often limited by safety concerns.

## Length of stay and clinical implications

Interestingly, a slight but statistically noteworthy increase in hospital length of stay was observed following protocol implementation, with the mean duration increasing from 10.45 nights (SD = 8.41) pre-protocol to 11.21 nights (SD = 7.68) post-protocol, the independent t-test indicated a mean increase of 0.76 nights, with a 95% CI [−0.90, −0.62] and p < 0.0001. While this might initially appear counterintuitive given improved analgesia and opioid sparing, several potential explanations exist. These include changes in institutional discharge policies unrelated to the pain management protocol, case mix variations in patient complexity, trends in cardiac surgery practice during the study period, or unmeasured confounding factors. This finding contrasts with previous trials demonstrating that multimodal analgesia protocols are typically associated with reduced length of stay (Williams et al., 2019; Li et al., 2018). Further investigation with prospective design and more rigorous control for confounding variables is needed to clarify this unexpected finding and determine whether it represents a true effect of the protocol or reflects limitations in the retrospective design.

## Limitations

Several limitations should be acknowledged. The retrospective, single-center design may limit generalizability to other institutions with different patient populations, practice patterns, and healthcare systems. The unequal group sizes reflect real-world implementation constraints but may introduce statistical limitations and reduce power to detect differences in certain subgroup analyses. With respect to surgical urgency, the current dataset does not include a structured variable distinguishing elective from emergent procedures. Based on institutional practice patterns, the majority of cardiac surgeries performed during the study period were elective; however, because urgency was not systematically coded in the electronic medical record, this distribution cannot be quantified retrospectively with high precision. Similarly, although comorbidities such as diabetes, chronic kidney disease, and other chronic conditions are documented at the individual patient level, these variables were not extracted during the initial dataset construction and are therefore unavailable for comparative analysis in the present retrospective cohort. Designed prospective study with more granular comorbidity characterization would enhance external validity.

Pain assessments were based on routine clinical documentation with inherent variability in measurement techniques, documentation practices, and potential reporting bias. Although we adjusted for confounders where possible, unmeasured factors such as changes in surgical technique, anesthetic management, or other aspects of perioperative care might influence outcomes.

Additionally, while no serious safety concerns emerged in this study, rare adverse events such as agranulocytosis cannot be definitively ruled out based on a single-center retrospective analysis with limited sample size. The relatively short follow-up period precluded assessment of long-term outcomes including chronic post-sternotomy pain, persistent opioid use, and delayed complications. In our dataset, documentation of opioid-related clinical adverse effects (e.g., nausea/vomiting, constipation, delirium, respiratory depression) was incomplete and therefore not suitable for reliable analysis. It should also be noted that chest wall regional analgesia techniques (such as paravertebral blocks or erector spinae plane blocks) are not routinely performed in our institution for cardiac surgery, which may limit the applicability of our findings to centers where regional anesthesia is a standard component of multimodal analgesia protocols. The relative contribution of metamizole within a more comprehensive multimodal approach that includes regional techniques remains to be determined.

## Conclusion and study highlights

In conclusion, our findings provide real-world evidence that scheduled metamizole as part of a multimodal analgesia protocol effectively reduces opioid consumption after cardiac surgery while maintaining adequate pain control and without compromising renal or hematologic safety.

These results support the integration of around-the-clock metamizole into postoperative analgesia and opioid sparing strategies in cardiac surgical populations where this medication is available and approved for use, particularly in patients for whom traditional non-opioid options may be contraindicated. Given the limitations of retrospective observational studies, prospective randomized controlled trials are warranted to confirm these findings and establish optimal dosing strategies, define appropriate safety monitoring protocols, and assess long-term outcomes including chronic pain, quality of life, and functional recovery. A prospective randomized trial would yield higher-quality evidence, incorporating a design powered on the observed between-group difference in opioid consumption (mean difference of MME), inclusion of patient-centered secondary outcomes (such as opioid-related adverse effects, NRS thresholds, and length of stay), and predefined surveillance for the hematologic and renal safety of metamizole. Future research should also investigate the comparative effectiveness of metamizole versus other non-opioid analgesics in cardiac surgery, evaluate its role in specific subpopulations (such as those with chronic kidney disease, advanced age, or baseline hematologic abnormalities), examine cost-effectiveness within comprehensive care pathways, and assess its integration with regional anesthesia techniques. The potential inclusion of metamizole in standardized ERAS protocols for cardiac surgery should be formally evaluated through multicenter collaborative research, particularly in regions where this medication is approved for clinical use. Such evidence would help inform international guidelines and optimize perioperative postoperative pain management strategies. Ongoing pharmacovigilance remains essential to continue monitoring the safety profile of metamizole in this vulnerable population, with particular attention to rare but serious adverse events and their early detection and management.

## Data Availability

The raw data supporting the conclusions of this article will be made available by the authors, without undue reservation.

## References

[B1] Aguerreche C. Cadier G. Beurton A. Imbault J. Leuillet S. Remy A. (2021). Feasibility and postoperative opioid sparing effect of an opioid-free anaesthesia in adult cardiac surgery: a retrospective study. BMC Anesthesiol. 21 (1), 166. 10.1186/s12871-021-01382-4 34082712 PMC8173983

[B2] Andrade C. (2020). Metamizole and agranulocytosis: what are the risks? J. Clin. Psychiatry 81 (4), 20f13699. 10.4088/JCP.20f13699 32726522

[B3] Anekar A. A. Hendrix J. M. Cascella M. (2023). WHO analgesic ladder. Treasure Island, FL: StarPearls. 32119322

[B4] Bachmann F. Duthaler U. Meyer Zu Schwabedissen H. E. Puchkov M. Huwyler J. Haschke M. (2021). Metamizole is a moderate cytochrome P450 inducer Via the constitutive androstane receptor and a weak inhibitor of CYP1A2. Clin. Pharmacol. Ther. 109 (6), 1505–1516. 10.1002/cpt.2141 33336382 PMC8247900

[B5] Bignami E. Castella A. Pota V. Saglietti F. Scognamiglio A. Trumello C. (2018). Perioperative pain management in cardiac surgery: a systematic review. Minerva Anestesiol. 84 (4), 488–503. 10.23736/S0375-9393.17.12142-5 29027773

[B6] Bignami E. Castella A. Pota V. (2020). Perioperative pain medicine in cardiac surgery: a consensus statement from the Italian society of anesthesia, analgesia, resuscitation and intensive care (SIAARTI). Ann. Card. Anaesth. 23 (4), 380–393. 10.4103/aca.ACA_30_20 32687111

[B7] Blitz M. J. Rochelson B. Prasannan L. Stoffels G. J. Pappas K. Palleschi G. T. (2022). Scheduled versus as-needed postpartum analgesia and oxycodone utilization. J. Matern. Fetal Neonatal Med. 35 (6), 1054–1062. 10.1080/14767058.2020.1742318 32193961

[B8] Chatzimanouil M. K. T. Müller K. Tzovaras D. (2023). Metamizole-induced agranulocytosis (MIA): a mini-review. Front. Pharmacol. 14, 1227308. 10.3389/fphar.2023.1227308

[B9] Dizner-Gołąb A. Kosson D. Lisowska B. (2025). Metamizole (dipyrone) for multimodal analgesia in postoperative pain in adults. Palliat. Med. Pract. 19 (1), 58–72. 10.5603/pmp.98765

[B10] Dowell D. Ragan K. Jones C. Baldwin G. Chou R. for the Centers for Disease Control and Prevention (2022). CDC clinical practice guideline for prescribing opioids for pain — united States, 2022. MMWR Recomm. Rep. 71 (3), 1–95. Available online at: https://www.cdc.gov/mmwr/volumes/71/rr/rr7103a1.htm. 36327391 10.15585/mmwr.rr7103a1 PMC9639433

[B11] Echeverria-Villalobos M. Stoicea N. Todeschini A. B. Fiorda-Diaz J. Uribe A. A. Weaver T. (2020). Enhanced recovery after surgery (ERAS): a perspective review of postoperative pain management under ERAS pathways and its role on the opioid crisis in the United States. Clin. J. Pain 36 (3), 219–226. 10.1097/AJP.0000000000000792 31868759

[B12] El-Diasty M. M. Rodríguez J. Pérez L. Souaf S. Eiras S. Fernández A. L. (2024). Compartmentalization of the inflammatory response in the pericardial cavity in patients undergoing cardiac surgery. Int. J. Mol. Sci. 25 (24), 13720. 10.3390/ijms252413720 39769482 PMC11676150

[B13] EMA (2025). EMA_Prescribing_information_Drug label information _METAMIZOLE®.

[B14] Erlenwein J. (2016). Management of acute pain therapy: guidelines, recommendations and current practice in German hospitals. AINS 51 (1), 40–48. 10.1055/s-0041-101757 26863643

[B15] European Medicines Agency (2024). EMA recommends measures to minimise serious outcomes of known side effect with painkiller metamizole. Available online at: https://www.ema.europa.eu/en/news/ema-recommends-measures-minimise-serious-outcomes-known-side-effect-painkiller-metamizole.

[B16] FDA (2025). FDA_Prescribing_information_Drug label information _TYLENOL®.

[B17] Fleming I. O. Garratt C. Guha R. Desai J. Chaubey S. Wang Y. (2016). Aggregation of marginal gains in cardiac surgery: feasibility of a perioperative care bundle for enhanced recovery in cardiac surgical patients. J. Cardiothorac. Vasc. Anesth. 30 (3), 665–670. 10.1053/j.jvca.2016.01.017 27321791

[B18] Grant M. C. Isada T. Ruzankin P. Gottschalk A. Whitman G. Lawton J. S. (2020). Opioid-sparing cardiac anesthesia: secondary analysis of an enhanced recovery program for cardiac surgery. Anesth. Analg. 131 (6), 1852–1861. 10.1213/ANE.0000000000005164 32889848

[B19] Grant M. C. Chappell D. Gan T. J. Manning M. W. Miller T. E. Brodt J. L. (2023). Pain management and opioid stewardship in adult cardiac surgery: joint consensus report of the PeriOperative quality initiative and the ERAS cardiac society. J. Thorac. Cardiovasc Surg. 166 (6), 1695–1706.e2. 10.1016/j.jtcvs.2023.01.020 36868931

[B20] Gregory A. J. Arora R. C. Chatterjee S. (2024). Enhanced recovery after surgery (ERAS) cardiac turnkey order set for perioperative pain management in cardiac surgery: proceedings from the American association for thoracic surgery (AATS) ERAS conclave 2023. JTCVS Open 20, 77–109. 10.1016/j.xjon.2024.06.005 PMC11704536 39780778

[B21] Guimarães-Pereira L. Farinha F. Azevedo L. Abelha F. Castro-Lopes J. (2016). Persistent postoperative pain after cardiac surgery: incidence, characterization, associated factors and its impact in quality of life. Eur. J. Pain 20 (9), 1433–1442. 10.1002/ejp.866 26988335

[B22] Guinot P. G. Spitz A. Berthoud V. Ellouze O. Missaoui A. Constandache T. (2019). Effect of opioid-free anaesthesia on post-operative period in cardiac surgery: a retrospective matched case-control study. BMC Anesthesiol. 19 (1), 136. 10.1186/s12871-019-0802-y 31366330 PMC6668113

[B23] Inoue S. Miyoshi H. Hieda K. Hayashi T. Tsutsumi Y. M. Teishima J. (2021). Around-the-clock postoperative intravenous acetaminophen reduces rescue opioid use after robot-assisted radical prostatectomy. Sci. Rep. 11, 5174. 10.1038/s41598-021-84696-w 33664398 PMC7933238

[B24] Jeyaraman N. Migliorini F. Murugan S. Ramasubramanian S. Balaji S. Maffulli N. (2024). Metamizole in the management of musculoskeletal disorders: current concept review. J. Clin. Med. 13 (16), 4794. 10.3390/jcm13164794 39200936 PMC11355082

[B25] Kötter T. da Costa B. R. Fässler M. (2015). Metamizole-associated adverse events: a systematic review and meta-analysis. PLoS One 10 (4), e0122918. 10.1371/journal.pone.0122918 25875821 PMC4405027

[B26] Lako S. Dedej T. Nurka T. Ostreni V. Demiraj A. Xhaxho R. (2015). Hematological changes in patients undergoing coronary artery bypass surgery: a prospective study. Med. Arch. 69 (3), 181–183. 10.5455/medarh.2015.69.181-183 26261388 PMC4500299

[B27] Li M. Zhang J. Gan T. J. Qin G. Wang L. Zhu M. (2018). Enhanced recovery after surgery pathway for patients undergoing cardiac surgery: a randomized clinical trial. Eur. J. Cardiothorac. Surg. 54 (3), 491–497. 10.1093/ejcts/ezy100 29514224

[B28] Lindhardt R. B. Buhl S. Molke J. (2024). The impact of acute kidney injury on chronic kidney disease after cardiac surgery: a systematic review. J. Thorac. Cardiovasc Surg. 168 (2), 421–432. 10.1016/j.jtcvs.2023.10.052

[B29] Loria R. El Moheb M. Khabbaz K. (2022). Enhanced recovery after cardiac surgery: a systematic review of pathways and outcomes. JTCVS Open 10, 92–105. 10.1016/j.xjtc.2022.08.007

[B30] Lui K. Kelly A. Williams C. (2023). Variation in prescribing for prevention of postoperative nausea/vomiting and pain following abdominal surgery: a retrospective audit. Health Sci. Rep. 6 (7), e1335. 10.1002/hsr2.1335 37324247 PMC10265722

[B31] Luz M. do Amaral Lopes S. A. V. Barreto B. B. (2025). Effectiveness of dipyrone (metamizole) in postoperative analgesia: a systematic review and meta-analysis. Trends Anaesth. Crit. Care 56, 101540. 10.1016/j.tacc.2025.101540

[B32] Makkad B. Heinke T. L. Sheriffdeen R. Meng M. L. Kachulis B. Grant M. C. (2025). Practice advisory for postoperative pain management of cardiac surgical patients: executive summary. A report from the society of cardiovascular anesthesiologists. J. Cardiothorac. Vasc. Anesth. 39 (1), 40–48. 10.1053/j.jvca.2024.09.005 39551694

[B33] McDonall J. Wilson J. Botti M. Hutchinson A. (2025). Patient experience of pain management following cardiac surgery: a mixed methods study. Pain Manag. Nurs. 26 (3), 271–281. 10.1016/j.pmn.2024.10.012 39542767

[B34] Othenin-Girard V. Osswald-Juilliard V. Levrat Q. (2025). Enhanced recovery after cardiac surgery protocols reduce opioid prescription at discharge: a before–after study. J. Clin. Med. 14 (5), 1768. 10.3390/jcm14051768 40095860 PMC11901073

[B35] Petermichl W. Ellmauer P. P. Benning A. Zeman F. Schmid C. Stadlbauer A. (2025). Impact of dipyrone administration on postoperative analgesia and aspirin effect in patients undergoing coronary artery bypass grafting: the randomized DipASA study. J. Cardiothorac. Vasc. Anesth. 39 (1), 121–130. 10.1053/j.jvca.2024.10.009 39490312

[B36] Rauseo M. Mirabella L. Carrideo A. A. Padovano F. P. Cantatore L. P. Vetuschi P. (2025). Opioid-sparing anesthesia in cardiac surgery: a meta-analysis. J. Cardiothorac. Vasc. Anesth. 39 (25), S1053–S1770. 10.1053/j.jvca.2025.06.040 40685295

[B37] Robich M. P. (2020). Prolonged cardiopulmonary bypass is associated with postoperative inflammatory activation and white blood cell elevation. J. Thorac. Cardiovasc Surg. 159 (5), e313–e315. 10.1016/j.jtcvs.2020.02.014 31982120

[B38] Scurt F. G. Nkuipou-Kenfack E. Schödel J. (2024a). Cardiac surgery–associated acute kidney injury. Kidney360 5 (6), 885–899. 10.34067/KID.0000000000000000 PMC11219121 38689404

[B39] Scurt F. G. Bose K. Mertens P. R. Chatzikyrkou C. Herzog C. (2024b). Cardiac surgery-associated acute kidney injury. Kidney360 5 (6), 909–926. 10.34067/KID.0000000000000466 38689404 PMC11219121

[B40] Squiccimarro E. Lorusso R. Alenizy K. (2022). Narrative review of the systemic inflammatory reaction to cardiac surgery and cardiopulmonary bypass. Front. Cardiovasc Med. 9, 863113. 10.3389/fcvm.2022.863113 PMC9303696 35061922

[B41] Tomidis Chatzimanouil M. K. Goppelt I. Zeissig Y. Sachs U. J. Laass M. W. (2023). Metamizole-induced agranulocytosis (MIA): a mini review. Mol. Cell Pediatr. 10 (1), 6. 10.1186/s40348-023-00160-8 37589909 PMC10435429

[B42] Wang Y. Bellomo R. (2017). Cardiac surgery-associated acute kidney injury: risk factors, pathophysiology and treatment. Nat. Rev. Nephrol. 13 (11), 697–711. 10.1038/nrneph.2017.119 28869251

[B43] Williams J. B. McConnell G. Allender J. E. Woltz P. Kane K. Smith P. K. (2019). One-year results from the first US-based enhanced recovery after cardiac surgery (ERAS cardiac) program. J. Thorac. Cardiovasc Surg. 157 (5), 1881–1888. 10.1016/j.jtcvs.2018.10.164 30665758

[B44] Yoon S. H. Ju J. W. Lee H. J. Kim J. Kim M. J. Park J. W. (2025). Development of the Korean enhanced recovery after surgery audit program (K-ERAS) and baseline compliance. Sci. Rep. 15, 27409. 10.1038/s41598-025-27409-8 40721595 PMC12304143

